# Supplementary material for: CD8+ T cell-derived Fgl2 regulates immunity in a cell-autonomous manner via ligation of FcγRIIB
Source: Nat Commun. 2024 Jun 20;15:5280. doi: 10.1038/s41467-024-49475-8 (PMC11190225; doi:10.1038/s41467-024-49475-8)
Supplement: Supplementary file 1 — Supplementary Information [file 41467_2024_49475_MOESM1_ESM.pdf]

CD8^{+} T cell-derived Fgl2 regulates immunity in a cell-autonomous manner via ligation of FcγRIIB

Supplementary Information

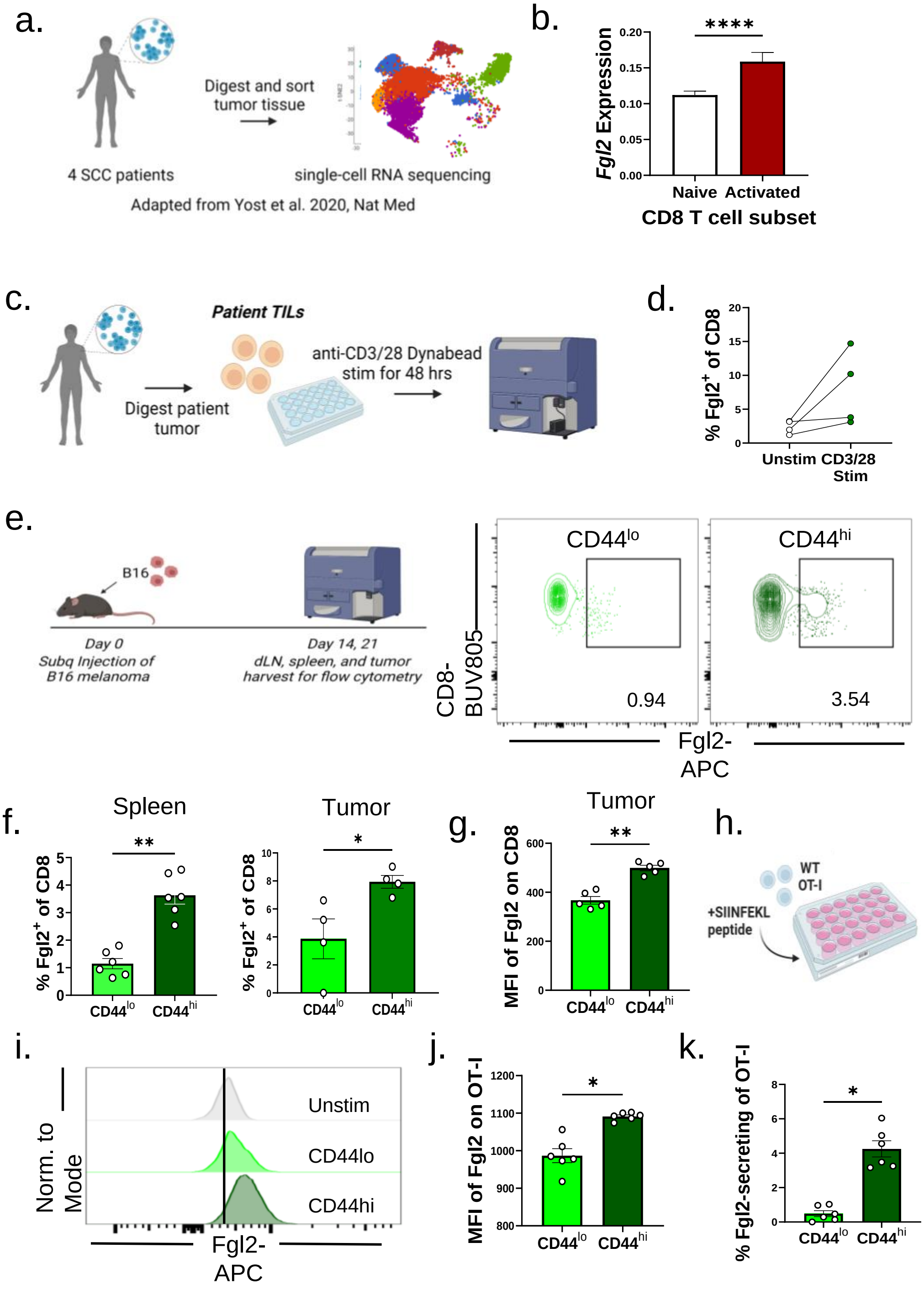

Supplementary Figure 1. *Fgl2* gene is expressed and Fgl2 protein is secreted by activated CD8$^+$ T cells in humans and mice. (A) Schematic and (B) summary data of *Fgl2* RNA expression on naïve vs. activated CD8$^+$ T cells at the tumor of four squamous cell carcinoma patients from a publicly available dataset deposited by Yost et al.[1] (C) Schematic and (D) summary data of Fgl2 protein expression on CD3$^+$CD8$^+$ TIL from digested melanoma patient tumor (n=4 patients) after a 48-hour CD3/28 Dynabead stimulation. (E) Schematic and representative flow plots as well as (F) summary data showing frequency of Fgl2$^+$ on CD44^{lo} vs. CD44^{hi} CD8$^+$ T cells stimulated with SIINFEKL peptide *ex vivo* from the spleen (n=6 mice) and tumor (n=4 mice) of B16-OVA challenged mice. (G) Mean fluorescence intensity of Fgl2 on *ex vivo* stimulated CD8$^+$ T cells from the tumor is also shown n=5 mice). Representative data from two independent experiments. (H) Schematic showing multiday *in vitro* stimulation of OT-I, (I) representative flow histograms and summary data showing (J) mean fluorescence intensity and (K) frequency of Fgl2$^+$ OT-I after three-day stimulation with SIINFEKL peptide compared to unstimulated controls. Representative data from two independent experiments, n=6 per group. Mann-Whitney non-parametric, unpaired two-sided test was used. The error bar in summary figures denotes mean ± SEM. *p<0.05 **p<0.01. Supplementary Figure 1A, 1C, 1E, and 1H was created with BioRender.com released under a Creative Commons Attribution-NonCommercial-NoDerivs 4.0 International license.

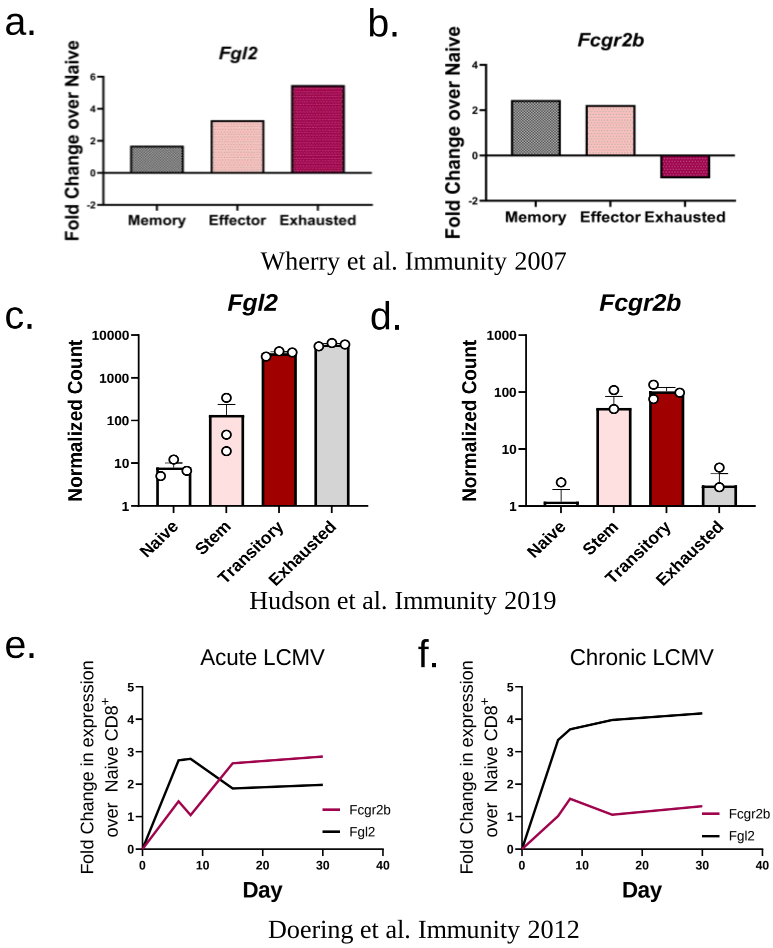

Supplementary Figure 2. *Fgl2* is expressed on differentiated CD8⁺ T cells in published datasets, notably when *Fcgr2b* expression is lowest. Bar graphs showing RNA expression of (A) *Fgl2* and (B) *Fcgr2b* in memory, effector, and exhausted CD8⁺ T cells as a fold change over naïve CD8⁺ during LCMV Clone 13 infection, both genes were differentially expressed in work by Wherry et al. in various CD8⁺ T cell subsets[2]. Average fold change shown of n=3 samples. Bar graphs showing RNA expression of (C) *Fgl2* and (D) *Fcgr2b* in naïve, stem-like, transitory, and exhausted CD8⁺ T cells as normalized read count during LCMV Clone 13 in work by Hudson et al.[3], n=3 samples per subset. Longitudinal RNA expression of *Fgl2* and *Fcgr2b* in gp33-specific CD8⁺ T cells as fold change over naïve CD8⁺ on days 6, 8, 15, and 30 post LCMV (E) Armstrong (acute) or (F) Clone 13 (chronic) viral infection in work by Doering et al.[4], average fold change is shown of n=4 samples per day per group. The error bar denotes mean ± SEM.

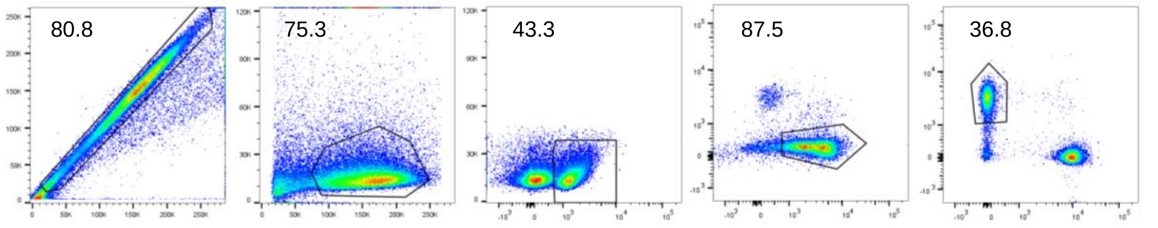

FSC-A/FSC-H → FSC-A/SSC-A → CD45/SSC-A → CD3/CD14&CD19 → CD4/CD8

Supplementary Figure 3. Flow cytometry gating strategy. Representative flow cytometry gating strategy is shown for the following experiments: Figure 1A-1E, Figure 2C-2G, 2I, 2J, 2L-2N, 2P-2R, Figure 3D-3I, Figure 4B-4E, 4G-4J, 4L-4Q, Supp. Data Fig. 1E-1K, and Supp. Data Fig. 3C-3G. First, events were gated on singlets via FSC-A/FSC-H. Lymphocytes were then gated using FSC-A/SSC-A followed by positive staining for the leukocyte marker CD45. $CD3^{+}$ $CD14^{-}CD19^{-}$ were next identified and subsequently $CD8^{+}$ $CD4^{-}$ T cells were gated. This gating strategy was used to exclude contaminating $CD14^{+}$ monocytes and $CD19^{+}$ B cells as these cell types express a high level of FcγRIIB. Subsequent gates of this gating strategy are shown in the appropriate figure.

Supplementary Table 1. Antibodies used in this study.

Antibody/Resource (anti-mouse)	Clone	Source
biotinylated CD16/32	2.4G2 (1:100)	BD Biosciences
biotinylated IgG2b kappa isotype control	27-35 (1:100)	BD Biosciences
CD4- BUV496	GK1.5 (1:100)	BD Biosciences
CD8- BUV805	53-6.7 (1:100)	BD Biosciences
CD3- BUV737	17A2 (1:100)	BD Biosciences
CD19- BV510	6D5 (1:200)	Biolegend
CD14- BV510	Sa14-2 (1:200)	Biolegend
CD44- APC-Cy7	IM7 (1:300)	Biolegend
CD62L- PE-Cy7	MEL-14 (1:100)	Biolegend
Thy1.1- BV711	OX-7 (1:100)	Biolegend
Thy1.2- PerCP	53-2.1 (1:100)	Biolegend
PD-1- PE-Dazzle, BV421	29F.1A12 (1:100)	Biolegend
Streptavidin- APC, BV711	N/A (1:200)	Biolegend
TIM3- BUV395, BV786	5D12 (1:100)	BD Biosciences
CD45.1- BV510	A20 (1:100)	Biolegend
CD45.2- BV605	104 (1:100)	Biolegend
Eomes- BUV395	X4-83 (1:50)	BD Biosciences
TCF7/TCF1-BV421	S33-966 (1:50)	BD Biosciences
Ki-67- PE, BV605	11F6 (1:100)	Biolegend
Fgl2- PE	6D9 (1:300)	Abnova

Supplementary Table 2. Demographic and clinicopathologic data for patients with advanced stage melanoma (n=4).

Analysis of FcγRIIB^{+} tumor-infiltrating lymphocytes (TIL)		
Variable	Parameter	No. of patients (%)
Sex	Female	3 (75)
	Male	1 (25)
Age	<65	3 (75)
	≥65	1 (25)
Race	White	3 (75)
	Unknown	1 (25)
	Non-White	0 (0)
Anatomic Site	Skin	3 (75)
	Lymph node	1 (25)
Stage	I	1 (25)
	II	0 (0)
	III	2 (50)
	Unknown	1 (25)
Treatment	Chemotherapy + Ipilumimab + Nivolumab	0 (0)
	Radiation + Ipilumimab + Nivolumab	0 (0)
	Nivolumab	1 (25)
	Pembrolizumab	1 (25)
	None	2 (50)

SUPPLEMENTARY REFERENCES

1 Yost, K. E. *et al.* Clonal replacement of tumor-specific T cells following PD-1 blockade. *Nat. Med.* **25**, 1251-1259 (2019). https://doi.org:10.1038/s41591-019-0522-3

2 Wherry, E. J. *et al.* Molecular Signature of CD8+ T Cell Exhaustion during Chronic Viral Infection. *Immunity* **27**, 670-684 (2007). https://doi.org:https://doi.org/10.1016/j.immuni.2007.09.006

3 Hudson, W. H. *et al.* Proliferating Transitory T Cells with an Effector-like Transcriptional Signature Emerge from PD-1⁺ Stem-like CD8⁺ T Cells during Chronic Infection. *Immunity* **51**, 1043-1058.e1044 (2019). https://doi.org:10.1016/j.immuni.2019.11.002

4 Doering, T. A. *et al.* Network analysis reveals centrally connected genes and pathways involved in CD8+ T cell exhaustion versus memory. *Immunity* **37**, 1130-1144 (2012). https://doi.org:10.1016/j.immuni.2012.08.021
