# Supplementary material for: CD8+ T cell-derived Fgl2 regulates immunity in a cell-autonomous manner via ligation of FcγRIIB
Source: Nat Commun. 2024 Jun 20;15:5280. doi: 10.1038/s41467-024-49475-8 (PMC11190225; doi:10.1038/s41467-024-49475-8)
Supplement: Supplementary file 3 — Reporting Summary [file 41467_2024_49475_MOESM3_ESM.pdf]

nature portfolio

Corresponding author(s): Mandy L. Ford

Last updated by author(s): May 13, 2024

Reporting Summary

Nature Portfolio wishes to improve the reproducibility of the work that we publish. This form provides structure for consistency and transparency in reporting. For further information on Nature Portfolio policies, see our Editorial Policies and the Editorial Policy Checklist.

Statistics

For all statistical analyses, confirm that the following items are present in the figure legend, table legend, main text, or Methods section.

n/a	Confirmed	
☐	☒	The exact sample size (*n*) for each experimental group/condition, given as a discrete number and unit of measurement
☐	☒	A statement on whether measurements were taken from distinct samples or whether the same sample was measured repeatedly
☐	☒	The statistical test(s) used AND whether they are one- or two-sided<br>*Only common tests should be described solely by name; describe more complex techniques in the Methods section.*
☒	☐	A description of all covariates tested
☐	☒	A description of any assumptions or corrections, such as tests of normality and adjustment for multiple comparisons
☐	☒	A full description of the statistical parameters including central tendency (e.g. means) or other basic estimates (e.g. regression coefficient) AND variation (e.g. standard deviation) or associated estimates of uncertainty (e.g. confidence intervals)
☐	☒	For null hypothesis testing, the test statistic (e.g. *F*, *t*, *r*) with confidence intervals, effect sizes, degrees of freedom and *P* value noted<br>*Give P values as exact values whenever suitable.*
☒	☐	For Bayesian analysis, information on the choice of priors and Markov chain Monte Carlo settings
☒	☐	For hierarchical and complex designs, identification of the appropriate level for tests and full reporting of outcomes
☒	☐	Estimates of effect sizes (e.g. Cohen's *d*, Pearson's *r*), indicating how they were calculated

Our web collection on statistics for biologists contains articles on many of the points above.

Software and code

Policy information about availability of computer code

Data collection	Libraries were validated by capillary electrophoresis on a TapeStation 4200 (Agilent), pooled at equimolar concentrations, and sequenced with PE100 reads on an Illumina NovaSeq 6000, yielding ~25 million reads per sample on average. Alignment was performed using STAR version 2.9.7a and transcripts were annotated using GRCm38_102. Transcript abundance estimates were calculated internal to the 20 STAR aligner using the algorithm of htseq-count.
Data analysis	Raw counts were analyzed in R using the DESeq258 version 1.41.12 and clusterProfiler59 4.8.2 packages from Bioconductor. Differentially expressed gene analyses were performed and differentially expressed genes were processed through GSEA.HALLMARK gene sets and C7 immunological signature gene sets from the Mouse Molecular Signatures Database (MSigDB) were used. Specifically, "HALLMARK_APOPTOSIS" and "GSE41867_MEMORY_VS_EXHAUSTED_CD8_TCELL_DAY30_LCMV_UP" are shown. A p-value <0.05 adjusted with the Benjamini-Hochberg correction was considered significant. Adjusted pvalue and enrichment scores are shown. No custom software that is not publicly available was utilized in this study. However, code is available upon request.

For manuscripts utilizing custom algorithms or software that are central to the research but not yet described in published literature, software must be made available to editors and reviewers. We strongly encourage code deposition in a community repository (e.g. GitHub). See the Nature Portfolio guidelines for submitting code & software for further information.

Data

Policy information about availability of data

All manuscripts must include a data availability statement. This statement should provide the following information, where applicable:

- Accession codes, unique identifiers, or web links for publicly available datasets
- A description of any restrictions on data availability
- For clinical datasets or third party data, please ensure that the statement adheres to our policy

The raw data is viewable in the NCBI SRA database under PRJNA1056506. The data generated in this study have been deposited in the GEO database and is available under the GEO database accession number GSE252556 available here, [https://www.ncbi.nlm.nih.gov/geo/query/acc.cgi?acc=GSE252556]). Source data are provided with this paper.

Research involving human participants, their data, or biological material

Policy information about studies with human participants or human data. See also policy information about sex, gender (identity/presentation), and sexual orientation and race, ethnicity and racism.

Reporting on sex and gender	This information has been collected and is outlined in Supplementary Data Table 2. Sex has been used as an identifier.
Reporting on race, ethnicity, or other socially relevant groupings	The terms "white" "unknown" and "non-white" were used to report, external variables were not controlled for.
Population characteristics	This information has been collected and is outlined in Supplementary Table 2.
Recruitment	For TIL studies, melanoma tumor tissues were collected (IRB #00095411), deidentified, and distributed by the Cancer Tissue and Pathology shared resource of Winship Cancer Institute of Emory University.
Ethics oversight	IRB board from Emory University. the study design and conduct complied with all relevant regulations regarding the use of human study participants and was conducted in accordance with the criteria set by the Declaration of Helsinki.

Note that full information on the approval of the study protocol must also be provided in the manuscript.

Field-specific reporting

Please select the one below that is the best fit for your research. If you are not sure, read the appropriate sections before making your selection.

[x] Life sciences [ ] Behavioural & social sciences [ ] Ecological, evolutionary & environmental sciences

For a reference copy of the document with all sections, see nature.com/documents/nr-reporting-summary-flat.pdf

Life sciences study design

All studies must disclose on these points even when the disclosure is negative.

Sample size	Samples sizes were determined by the resource equation model, 2-3 independent experiments were performed. Experimental repeats were successful.
Data exclusions	Outliers were detected using the ROUT method.
Replication	In each experiment, 3-4 mice were used per group and 2-3 independent experiments were performed.
Randomization	Animals were randomized to groups, mice of similar sex and age were used across groups.
Blinding	Groups were not blinded, ARRIVE guidelines were followed.

Reporting for specific materials, systems and methods

We require information from authors about some types of materials, experimental systems and methods used in many studies. Here, indicate whether each material, system or method listed is relevant to your study. If you are not sure if a list item applies to your research, read the appropriate section before selecting a response.

Materials & experimental systems

n/a	Involved in the study	
	☒	Antibodies
	☒	Eukaryotic cell lines
☒		Palaeontology and archaeology
	☒	Animals and other organisms
	☒	Clinical data
☒		Dual use research of concern
☒		Plants

Methods

n/a	Involved in the study	
☒		ChIP-seq
	☒	Flow cytometry
☒		MRI-based neuroimaging

Antibodies

Antibodies used	Antibodies were diluted at a 1:100 dilution unless stated otherwise next to the antibody.<br>Antibody/Resource, Clone, Source<br>biotinylated CD16/32 2.4G2 BD Biosciences<br>biotinylated IgG2b kappa isotype control 27-35 BD Biosciences<br>CD4- BUV496 GK1.5 BD Biosciences<br>CD8- BUV805 53-6.7 BD Biosciences<br>CD3- BUV737 17A2 BD Biosciences<br>CD19- BV510 6D5 Biolegend<br>CD14- BV510 Sa14-2 Biolegend<br>CD44- APC-Cy7 IM7 Biolegend (1:300)<br>CD62L- PE-Cy7 MEL-14 Biolegend<br>Thy1.1- BV711 OX-7 Biolegend<br>Thy1.2- PerCP 53-2.1 Biolegend<br>PD-1- PE-Dazzle, BV421 29F.1A12 Biolegend<br>Streptavidin- APC, BV711, PE N/A Biolegend (1:200)<br>TIM3- BUV395, BV786 5D12 BD Biosciences<br>CD45.1- BV510 A20 Biolegend<br>CD45.2- BV605 104 Biolegend<br>Eomes- BUV395 X4-83 BD Biosciences (1:50)<br>TCF7/TCF1-BV421 S33-966 BD Biosciences (1:50)<br>Ki-67- PE, BV605 11F6 Biolegend (1:50)<br>Fgl2- PE, APC 6D9 Abnova (1:300)
Validation	Antibodies were validated as mentioned by the manufacturer either through isotype control or genetic knockout verification.

Eukaryotic cell lines

Policy information about cell lines and Sex and Gender in Research

Cell line source(s)	The B16 melanoma cell line engineered to express the OVA epitope was provided by Dr. YangXin Fu, University of Texas Southwest, Dallas, TX. The parental B16F10 cell line used is from ATCC.<br>The Jurkat E6-1 immortalized T cell line was purchased from ATCC.
Authentication	The cell lines were authenticated by Emory Division of Animal Resources and ATCC
Mycoplasma contamination	Cell lines were tested were tested and found mycoplasm negative. As well as negative for the following pathogens: Corynebacterium bovis, Lymphocytic Choriomeningitis Virus, Lactate Dehydrogenase Elevating Virus,<br>Murine Adenovirus-1 &2, Murine Cytomegalovirus, Mouse Hepatitis Virus, Mouse Norovirus, Ectromelia Virus, Mouse Parvo Virus, Minute Virus of Mice, Epizootic Diarrhea of Infant Mice, Polyoma, Pneumonia Virus of Mice, Reovirus-3, Sendai Virus, Theiler's Mouse Encephalomyelitis Virus, Mycoplasma pulmonis, Mycoplasma spp by PCR assay.
Commonly misidentified lines (See ICLAC register)	No commonly misidentified cell lines were used.

Animals and other research organisms

Policy information about studies involving animals; ARRIVE guidelines recommended for reporting animal research, and Sex and Gender in Research

Laboratory animals	OT-I (Hogquist et al., 1994)51 transgenic mice were purchased from Taconic Farms and bred to Thy1.1+ (B6.PL-Thy1a/CyJ, Jackson Laboratory, Stock #000406) animals at Emory University. B6.Cg-Tcratm1Mom Tg(TcrLCMV)327Sdz/TacMmjax were purchased from Jackson Laboratory (Stock # 037394). EM:06078 Fcgr2b Fcgr2bB6null B6(Cg)-Fcgr2btm12Sjv/Cnbc (or Fcgr2b-/-) mice obtained under MTA with the Academisch Siekenhuis Leiden/Leiden University Medical Center and Dr. J.S. Verbeek. Cryopreserved embryos were

	shipped from the European Mutant Mouse Archive (EMMA) and re-derived at the Emory University Transgenic Mouse Core Facility. These mice were generated using embryonic stem cells from B6 mice. These Fcgr2b-/- mice made by Boross et al.52 were bred to OT-I transgenic mice at Emory University. Fgl2-/- mice were a kind gift of Dr. Gary Levy53, University of Toronto. Fgl2-/- mice were bred to OT-I or P14 Thy1.1+ TCR transgenic mice to generate Fgl2-/- OT-I mice and Fgl2-/- P14 mice. Mice were used at 8-12 weeks old. All animals were housed in specific pathogen-free animal facilities at Emory University. Control animals were bred separately in the same facility. Mice were housed at a humidity of ~55% and the dark/light cycle was 12h/12h.
Wild animals	No wild animals were involved in this study.
Reporting on sex	Animals were sex-matched for experiments and experiments were performed in male and female mice. No sex differences observed.
Field-collected samples	The study did not involve samples collected from the field.
Ethics oversight	Ethics oversight was given by IACUC approved by Emory University for animal studies.

Note that full information on the approval of the study protocol must also be provided in the manuscript.

Clinical data

Policy information about clinical studies
All manuscripts should comply with the ICMJEguidelines for publication of clinical research and a completedCONSORT checklist must be included with all submissions.

Clinical trial registration	No trial registration number
Study protocol	Not a trial so no study protocol available.
Data collection	The study design and conduct complied with the regulations on the use of human study participants and was conducted in accordance with the Declaration of Helsinki. This protocol was approved by Emory University's Institutional Review Board. For TIL studies, melanoma tumor tissues were collected (IRB no. 00095411), deidentified, and distributed by the Cancer Tissue and Pathology shared resource of Winship Cancer Institute of Emory University [demographic data shown in Supplementary Data Table 2 (n = 4)].
Outcomes	No primary or secondary outcome measures pre-defined.

Plants

Seed stocks	*Report on the source of all seed stocks or other plant material used. If applicable, state the seed stock centre and catalogue number. If plant specimens were collected from the field, describe the collection location, date and sampling procedures.*
Novel plant genotypes	*Describe the methods by which all novel plant genotypes were produced. This includes those generated by transgenic approaches, gene editing, chemical/radiation-based mutagenesis and hybridization. For transgenic lines, describe the transformation method, the number of independent lines analyzed and the generation upon which experiments were performed. For gene-edited lines, describe the editor used, the endogenous sequence targeted for editing, the targeting guide RNA sequence (if applicable) and how the editor was applied.*
Authentication	*Describe any authentication procedures for each seed stock used or novel genotype generated. Describe any experiments used to assess the effect of a mutation and, where applicable, how potential secondary effects (e.g. second site T-DNA insertions, mosiacism, off-target gene editing) were examined.*

Flow Cytometry

Plots

Confirm that:

- [x] The axis labels state the marker and fluorochrome used (e.g. CD4-FITC).
- [x] The axis scales are clearly visible. Include numbers along axes only for bottom left plot of group (a 'group' is an analysis of identical markers).
- [x] All plots are contour plots with outliers or pseudocolor plots.
- [x] A numerical value for number of cells or percentage (with statistics) is provided.

Methodology

Sample preparation	For tumor-infiltrating lymphocyte (TIL) experiments, patient tumor tissue was collected and dissociated using the Human Tumor Dissociation Kit (Miltenyi) and GentleMACS Octo Dissociator (Miltenyi) according to the manufacturer's instructions. Cells were activated in vitro using 15 µl/ml anti-CD3/28-coated Dynabeads (Thermofisher) in R10 for 48 hours at 37°C and 5% CO2. Flow staining was performed using the antibodies listed in Extended Data Table 1. Fgl2 staining was assessed via staining with Fgl2-APC antibody conjugated with Lightning Link technology.

For tumor experiments, spleen, draining lymph node (right inguinal proximal to tumor), and tumors of mice were processed to cell suspensions, blood underwent red blood cell (RBC) lysis prior to staining. For viral experiments, spleen, brachial and axillary lymph nodes were processed to cell suspensions, blood underwent RBC lysis prior to staining. The samples were then stained for surface markers prior to permeabilization for transcription factor staining using the antibodies listed in Supplementary Data Table 1. Cells were permeabilized using a FoxP3/transcription factor kit (Invitrogen). For Fgl2 cytokine staining of OT-I, splenocytes were ex vivo stimulated at 37°C for 4 hours with 10 nM OVA257-264 (SIINFEKL) peptide (B16-OVA) and 10 µg/mL GolgiPlug (BD Biosciences). For Fgl2 cytokine staining of P14 cells, splenocytes were ex vivo stimulated at 37°C for 4 hours with 0.4 µg/ml LCMV-gp33-41 (KAVYNFATM) peptide and 10 µg/mL GolgiPlug (BD Biosciences). After 4 hours, cells were processed and stained for intracellular markers using antibodies listed in Supplementary Data Table 1. Fgl2 antibody (Abnova) were conjugated to fluorophore with Lightning Link technology (or isotype) and validated using Fgl2-/-splenocytes.

Instrument	Experiments were run using Fortessa and LSR II flow cytometers as well as an Aria sorter.
Software	Flow cytometry data was analyzed using FlowJo (Tree Star) and Prism (GraphPad Software). Absolute cell numbers were calculated using CountBright Beads (Life Technologies) according to the manufacturer's instructions.
Cell population abundance	Purity of >95% for post-sort populations on Aria Cell Sorter was determined from a check of sample post-sort while on cell sorter.
Gating strategy	A gating strategy is in the supplementary information as Supp. Data Figure 3. For most experiments, the following gating strategy was employed: FSC-H by FSC-A to eliminate non-singlet events, then FSC-A by SSC-A to gate on lymphocytes followed by a CD45 gate, then CD14/19 by CD3. On CD3 population, subsequent events were visualized on CD4 by CD8 axes. Afterwards, CD8 by congenic marker (either CD45.1 or Thy1.1) was plotted, followed by phenotypic and functional analyses on this population. The list of experiments for which this gating strategy was employed is listed in the figure legend of Supp. Data Fig. 3 while the subsequent appropriate gating is shown in the appropriate figure.

☒ Tick this box to confirm that a figure exemplifying the gating strategy is provided in the Supplementary Information.
